# Improving risk equalization using information on physiotherapy diagnoses

**DOI:** 10.1007/s10198-017-0874-x

**Published:** 2017-02-09

**Authors:** Frank Eijkenaar, René C. J. A. van Vliet

**Affiliations:** Institute of Health Policy and Management, Erasmus University Rotterdam, Burgemeester Oudlaan 50, 3000 DR Rotterdam, The Netherlands

**Keywords:** I11, I13, G22, Health insurance, Risk equalization, Physiotherapy diagnoses, Claims data

## Abstract

**Background:**

Worldwide, risk-equalization (RE) models in competitive health insurance markets have evolved from simple demographic models to sophisticated models containing diagnosis and pharmacy-based indicators of health. However, these models still have important imperfections; adding information on (diagnoses of) physiotherapy treatment may further improve RE-models. Therefore, a new risk-adjuster based on physiotherapy costs in the prior year was introduced in the Dutch RE-model of 2016.

**Methods:**

Physiotherapy claims-data (2012) and administrative data on costs and risk-characteristics (2013) for 94% of the Dutch population (*N* = 15.8 million) are used to evaluate the current risk-adjuster based on physiotherapy costs and to assess the effects of replacing it by different modalities of a risk-adjuster based on physiotherapy diagnoses. Of the 89 diagnoses in the claims-data, 62 are dropped because they relate to temporary health problems. The 27 retained diagnoses are added to the Dutch model in 4 modalities: 27 separate risk-classes, 9 diagnosis-clusters based on main pathology category, 4 diagnosis-clusters based on residual costs, and the 4 clusters of modality 3 interacted with age.

**Results:**

Although the cost-based risk-adjuster improves the model’s predictive power and removes the average undercompensation (€919) for enrollees with physiotherapy costs in the prior year, it is outperformed by all 4 diagnosis-based modalities. Of these modalities, modality 3 is preferred based on its simplicity and comparable predictive power.

**Conclusions:**

Adding information on physiotherapy can further improve the performance of sophisticated RE-models. Regarding the Dutch model, a risk-adjuster containing 4 risk-classes for clustered diagnoses based on residual costs is the preferred modality.

**Electronic supplementary material:**

The online version of this article (doi:10.1007/s10198-017-0874-x) contains supplementary material, which is available to authorized users.

## Introduction

In an increasing number of competitive health insurance markets, risk equalization (RE) is used as a mechanism that provides insurers with a compensation for each enrolled person based on expected costs and adjusted for individual risk characteristics. As a result, insurers receive a higher payment for high-risk enrollees (e.g. the elderly and chronically ill) than for low-risk enrollees (e.g. the young and healthy). In the presence of premium-rate restrictions, the goal of RE is to reduce incentives for risk selection while maintaining insurers’ incentives for promoting efficiency of care [1].

Different forms of RE have been implemented in Europe [2–6] and the US [7, 8]. In the last decades, RE-models have developed from simple demographic models to sophisticated models using diagnosis and pharmacy-based indicators of health [3, 4, 7, 8]. For example, the Dutch RE-model for curative somatic care now contains about 160 risk-classes, almost half of which can be considered direct proxies for health because they are based on diagnoses or prior utilization related to chronic illness [4]. However, even these sophisticated models have important imperfections and result in undercompensations for individuals with rare diseases, (functional) disability, multi-morbidity, and poor self-reported health. For example, the Dutch RE-model of 2015 results in a statistically significant average undercompensation of €378 for the subgroup of individuals who judge their own health as ‘alright’, ‘poor’, or ‘very poor’ (about 20% of the population) [9]. Undercompensations such as these provide insurers with incentives for risk selection, which is undesirable because it may reduce quality of care, efficiency of care, and solidarity between healthy and chronically ill people [10]. Even in the presence of regulations such as open enrollment, no exclusion of preexisting conditions, and standardized insurance coverage, many (subtle) forms of risk selection are possible [11].

A potentially viable option for further improving RE-models is to make use of information on (diagnoses from) treatments in primary care. Although current sophisticated RE-models make extensive use of diagnosis information, this information is typically only related to specific hospital treatments [3, 4, 7, 8]. With this in mind, several improvements have been proposed for the Dutch RE-model, one of which is a risk-adjuster based on the prior use of physiotherapy [9]. The rationale of this adjuster is threefold. First, the RE-model 2015 results in an average undercompensation of about €1000 for the subgroup of almost 400,000 adults with physiotherapy costs under the basic insurance in the prior year [12]. Second, under the Dutch basic insurance, coverage for physiotherapy for adults starts at the 21st reatment (provided there is a referral from a physician who sets the diagnosis) and is limited to specific chronic conditions (see table, supplemental digital content 1). For individuals age 17 and younger, all treatments related to these conditions are covered, in addition to a maximum of 18 treatments for other conditions. Thus, a risk-adjuster based on the use of physiotherapy could potentially reduce undercompensations for chronically ill people. Finally, medical experts indicated that undercompensated groups of people with rare genetic diseases often make use of physiotherapy.

Therefore, a dummy-variable for yes/no physiotherapy costs in the prior year was added to the Dutch RE-model 2016 under the name ‘physiotherapy utilization groups’ (PUGs). For practical reasons (e.g. the difference in coverage), the PUGs only apply to adults. Although the PUGs removed the undercompensation for the subgroup with physiotherapy costs in the prior year, this risk-adjuster has several disadvantages [12]. First, since it is based on prior costs, it is ‘endogenous’ and reduces insurers’ incentives for promoting efficiency of care. Second, in the prior year’s cost-data it is not possible to distinguish between ‘permanent’ chronic conditions for which coverage exists regardless of the duration of the treatment, and ‘temporary’ conditions covered for a limited treatment duration only (see table, supplemental digital content 1). In RE, however, risk-adjusters ideally relate only to chronic conditions (not to temporary health problems) because the aim is to compensate insurers for *predictable* cost variation. Third, as the risk-adjuster is only based on yes/no costs in the prior year, it does not take the severity of the underlying diagnosis into account. Finally, the specific characteristics of the prior year’s cost data preclude inclusion of children in the risk-adjuster.

These problems may be overcome by using information on diagnoses of physiotherapy treatment (instead of on prior costs) [12]. The resulting adjuster would be a more direct proxy for health and similar to the diagnosis-based cost groups (DCGs) in the Dutch RE-model, in which enrollees are classified into risk-classes using diagnoses from hospital treatments [13, 14]. At the time the PUGs were developed, data on physiotherapy diagnoses were not available for research purposes. However, at the end of 2015, when a new research cycle for improving the RE-model of 2016 started, these data came available.

In this paper, using physiotherapy claims data of 2012 and administrative data on costs and risk characteristics of 2013 for (almost) the entire Dutch population, we evaluate the PUGs and assess the effects of replacing the PUGs by different modalities of an alternative risk-adjuster labeled ‘physiotherapy-diagnosis groups’ (PDGs). Specifically, our goal is threefold: (1) to study the possibilities of improving RE using information on (the use of) physiotherapy (2) to develop different modalities of a risk-adjuster based on physiotherapy diagnoses and (3) to assess the effects of replacing the PUGs by these modalities in terms of key criteria for adequate risk-adjusters, including predictive power, validity, and stability [1].

### The Dutch basic health insurance system

Since 2006, Dutch residents have been obliged to purchase basic health insurance coverage for a standardized benefits package. In 2016, this basic insurance scheme comprises about 70% of total healthcare spending and is characterized by a form of managed competition in which insurers compete on price and quality and enrollees can switch annually among insurers. The scheme comprises three main financial streams: a community-rated premium from adult enrollees to insurers, an income-related contribution from enrollees to the RE-fund (a national account from which the RE-payments to insurers are financed), and RE-payments between insurers and the RE-fund. The RE-payments are based on the expected costs of insurers’ enrollees in year *t*, which are based on the coefficients of an OLS-regression of costs on risk characteristics using data from *t*-3 (see table, supplemental digital content 2, for the coefficients of the Dutch RE-model of 2016 with and without the PUGs).

The Dutch RE-model 2016 contains the following ten risk-adjusters: age/gender (40 risk-classes), region (10 risk-classes based on clusters of zip-codes), socioeconomic status (17 risk-classes based mainly on income, interacted with age), source of income (24 risk-classes for self-employed, students, higher-educated, and people with social security benefits; interacted with age), pharmacy-based cost groups (PCGs, 30 risk-classes based on prior use of medication prescribed for chronic illnesses), DCGs (15 risk-classes based on diagnoses from hospital treatments in the prior year), durable medical equipment cost groups (DMECGs, 4 risk-classes based on prior use of durable medical equipment), multi-year high cost groups (MYHCGs, 6 risk-classes based on high costs in the prior 3 years), PUGs (1 risk-class), and 4 interaction risk-classes. In this model, the PCGs, DCGs, DMECGs and MYHCGs can be considered as ‘morbidity-based’ and thus as direct proxies for health.

In addition, the model contains 2 risk-adjusters based on the costs of home care and of geriatric revalidation in the prior year. However, both adjusters are excluded here because (1) the Dutch Minister of Health aims to abolish these ‘endogenous’ risk-adjusters ultimately by the end of 2018 [15] and (2) these adjusters may have confounding effects on the results.

## Methods

Two main datasets are used for this study. First, we use a dataset containing all basic health insurance claims (*N* = 14.4 million) for physiotherapy treatments provided to about 700,000 patients in 2012. Importantly, the claims contain a diagnosis-code based on a classification system that enables the physiotherapist to register the body-location and the pathology of the diagnosis using a 4-digit coding. Digits 3 and 4 refer to the pathology, with digit 3 being the main category (e.g. neurological condition) and digit 4 the subcategory (e.g. paraplegia). For this study, only digits 3 and 4 (henceforth: diagnosis) are relevant because for a given pathology, it is highly unlikely that a difference in body-location involves a difference in health. In total, 89 different diagnoses can be discerned in the claims data.

The second dataset contains administrative, individual-level data on costs and risk characteristics in 2013 for the entire Dutch population (*N* = 16.9 million). This dataset was actually used for calculating the RE-payments for Dutch insurers for 2016, and came from various administrative sources. Costs comprise the total costs for curative somatic care under the basic health insurance of 2016 (cost-level 2013). As is customary in the Dutch RE-model, costs of enrollees were annualized and weighted for each enrollee for the duration of the insurance contract in 2013.

Given these datasets, we used a 7-step procedure to address the research objectives:

1. Delete unusable/incorrect claims. We deleted physiotherapy claims with an unknown diagnosis-code and/or a negative claim-amount. In addition, we deleted clearly invalid claims (e.g. child physiotherapy claims submitted for adults) and all claims pertaining to the enrollees of 3 insurers because these insurers submitted incomplete or no claims. As a result of these selections, the number of patients with a physiotherapy diagnosis reduced to approximately 675,000.
2. Merge the cleaned claims data with the administrative data. Using a unique (anonymous) identification key, we merged the 2012 claims data with the 2013 administrative data on the individual enrollee-level. As a result of the first step, the number of enrollees in the administrative data reduced from 16.9 million to 15.8 million.
3. Estimate the Dutch RE-model 2016 by OLS using 2013-costs as the dependent variable and the above mentioned risk-adjusters *excluding* the PUGs as the explanatory variables, and calculate the predicted costs for each enrollee (see table, supplemental digital content 2, for the coefficients of this model).
4. Calculate the mean (residual) costs for each of the 89 diagnoses (see table, supplemental digital content 3). Mean residual costs are calculated by subtracting for each enrollee the predicted costs from the actual costs and averaging the result to the diagnosis-level.
5. Delete diagnoses related to temporary health problems. Sixty-two diagnoses related to conditions not covered for adults (e.g. respiratory infections) and/or to conditions with limited treatment duration (e.g. fractures) were dropped. These diagnoses have a relatively low prevalence and/or low residual costs (see table, supplemental digital content 3), implying limited predictive power compared to the 27 retained diagnoses. In this step, we were assisted by a team of medical professionals, experts from health insurance companies, and experts from the Dutch Ministry of Health.
6. Construct 4 PDG-modalities based on the retained 27 diagnoses: (1) the 27 diagnoses as separate risk-classes (2) the diagnoses clustered in 9 risk-classes based on main pathology category (3) the diagnoses clustered in 4 risk-classes based on residual costs, and (4) the 4 risk-classes from modality 3 interacted with age (0–17, 18–64, 65+). For modality 3, diagnoses were sorted on descending residual costs, in such a way that patients with multiple diagnoses are classified in the applicable diagnosis with the highest residual costs. Using Ward’s hierarchical clustering method [16], diagnoses were then clustered into 4 mutually exclusive groups based on homogeneity in residual costs, taking into account the diagnoses’ standard deviation and prevalence and in such a way that the differences among the diagnosis-clusters in mean residual costs is maximized. The rationale for modality 4 is that research shows that age may play a role when predicting healthcare costs using information on physiotherapy utilization [12].
7. Estimate (by OLS) 5 additional versions of the Dutch RE-model of 2016 and compare the results. These versions are the RE-model 2016 with the current PUGs and the RE-model 2016 with the PUGs replaced by 1 of the 4 PDG-modalities.

Assisted by a team of medical professionals and experts from health insurance companies, we assessed the validity of the 2012 claims data using (1) administrative data on individual-level costs for different types of primary care (including physiotherapy) for 2012 and 2013, and (2) physiotherapy claims data of 2013. Based on additional analyses on these data (see figures, supplemental digital content 4), the validity could be confirmed and did not lead to any further modifications or selections.

## Results

### Descriptive statistics

Table 1 shows descriptive statistics for all enrollees, for enrollees with physiotherapy costs in 2012, and for three groups of enrollees with a physiotherapy diagnosis in 2012. For (adult) enrollees with physiotherapy costs in 2012, total costs are on average €908 higher than for enrollees (of all ages) with at least 1 of the 27 diagnoses in 2012. However, residual costs are €434 higher in the latter group, which is particularly notable in view of the €238 *lower* residual costs for enrollees (of all ages) with at least 1 of the 89 *original* diagnoses as compared to enrollees with physiotherapy costs in 2012.

**Table 1:** Descriptive statistics for the year 2013

	All enrollees in merged dataset	Enrollees with physiotherapy costs in 2012 (i.e. PUG > 0)	Enrollees with ≥1 of 89 original diagnoses in 2012	Enrollees with ≥1 of 62 dropped diagnoses in 2012	Enrollees with ≥1 of 27 selected diagnoses in 2012
Included age groups	All ages	Ages 18+ only	All ages	All ages	All ages
*N* (weighted)^a^	15,523,485	312,282	664,139	377,797	286,342
Costs in € [mean (st. dev.)]	2162 [7927]	8836 [17,065]	5155 [14,099]	3054 [8702]	7928 [18,645]
Residual in € [mean (st. dev.)]^b^	0 [6850]	919 [14,496]	681 [11,708]	172 [7312]	1353 [15,704]
Age (mean)	40.8	61.0	34.7	33.9	35.9
Men (%)	49.3	37.2	45.4	42.6	49.1
Classified in a PCG (%)	19.6	55.6	28.9	21.8	38.4
Classified in a DCG (%)	9.4	41.8	22.2	17.3	28.7
Classified in a DMECG (%)	0.9	5.4	2.8	1.5	4.5
Classified in a MYHCG (%)	5.9	47.7	24.7	15.6	36.6
Classified in a PCG, DCG, DMECG, or MYHCG (%)	24.6	73.9	40.6	32.1	51.8

^a^ *N* = the number of enrollees weighted by the duration of enrollment in 2013

^b^Residual costs based on Dutch RE-model 2016 without the PUGs as a risk adjuster. Because the model is estimated by OLS, the average residual for all enrollees equals 0 (by definition)

The bottom row of Table 1 shows that almost 52% of enrollees with the 27 diagnoses are classified into one or more risk-classes of the current ‘morbidity-based’ risk-adjusters. This implies that any version of a risk-adjuster based on the 27 diagnoses will show considerable overlap with these risk-adjusters. This overlap, however, is substantially less compared to the current PUGs (see also table, supplemental digital content 5).

As a result of dropping 62 diagnoses, the (weighted) number of patients with at least 1 diagnosis decreased from 664,139 to 286,342. As shown in the last two columns of Table 1, mean residual costs for the dropped diagnoses are almost eight times lower than for the 27 retained diagnoses. Thus, the dropped diagnoses either have a low prevalence, low residual costs, or both, confirming the adequacy of basing the PDG-modalities on the 27 diagnoses.

### Stability over time

**Fig. 1:**
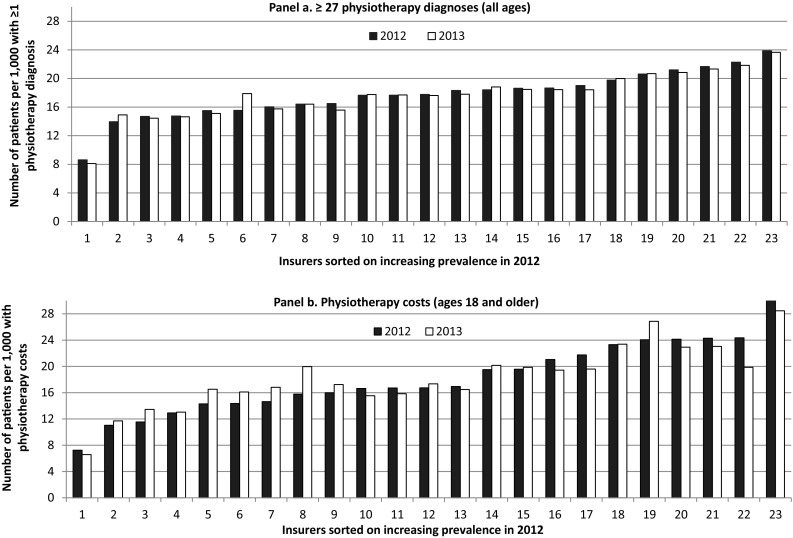
Number of patients per 1000 with at least one of 27 physiotherapy diagnoses (*panel a*) or with physiotherapy costs (*panel b*) for 23 risk-bearing insurers, for 2 years. In both panels, the numbers for the year 2012 are adjusted for the overall change in prevalence from 2012 to 2013

Ideally, the prevalence of a risk-adjuster is stable at the individual insurer-level; large fluctuations over time may indicate problems or differences in claims registration among insurers [1]. Figure 1 shows the prevalence of the 27 diagnoses and the PUGs, respectively, at the level of the 23 insurers, for 2 years. Figure 1 indicates that a risk-adjuster based on the 27 diagnoses will be more stable at the insurer-level than the PUGs (based on physiotherapy costs). In panel a, the mean absolute change from 2012 to 2013 is 0.28 per 1000 patients. In panel b, this is 1.17, while the overall prevalence is only 9% higher compared to panel A.

In an additional analysis, we examined the changes in prevalence at the diagnosis-level (see figure, supplemental digital content 6). The changes are very small, especially in comparison to the common year-to-year changes in the prevalence of the current PCGs and DCGs [17].

### PDG-modalities

Table 2 presents information for the PUGs and for 3 of the 4 PDG-modalities (for clarity of presentation, modality 4 is excluded in this table). For modality 1 and 2, summation of the (weighted) number of patients yields 289,375, about 3000 more than the 286,342 patients in Table 1. This means that only 1% of enrollees have multiple diagnoses, so classifying enrollees in the diagnosis with the highest residual costs, as done in all modalities when estimating the coefficients, will have very limited consequences for the predictive power.

**Table 2:** Key statistics for three PDG-modalities for the year 2013 (costs and coefficients in €)

Code/cluster	Description	*N* ^a^	Mean costs	Mean residual costs^b^	Estimated coefficient^c^
PUG					
	Physiotherapy costs in 2012	312,282	8836	919	1008
PDG-modality 1					
Code 00	Amputation	2536	15,603	1737	1971
Code 10	Aseptic bone necrosis	375	1377	354^ns^	423
Code 11	Disorders of the vertebral column/pelvis	19,344	2333	355	435
Code 12	Congenital disorders of the skeleton	5895	5250	1235	1334
Code 13	Ossification disorder	1107	4311	473	567
Code 14	Inflammation of/tumors in the skeleton	676	16,476	5948	6125
Code 28	Sudeck’ a(dys)trophy	4692	8351	1043	1173
Code 39	Status after burn wounds	149	7063	1732	1889
Code 46	Disorders of the lymphatic system/edema	21,578	9120	399	471
Code 51	Congenital disorders of respiratory system	566	35,374	11,253	12 049
Code 54	Chronic obstructive pulmonary disease	23,308	14,470	1840	2117
Code 56	Interstitial lung disorder including sarcoïdosis	920	14,792	3767	3534
Code 65	Other hereditary disorders	460	35,808	22,767	23,014
Code 69	Tumors without surgery	3273	17,060	1915	2058
Code 70	Peripheral nerve disorder	6404	9791	1819	1918
Code 71	Cerebellar disorders	6755	11,562	1068	1281
Code 72	Cerebrovascular accident/central paresis	27,049	13,049	2566	2773
Code 73	MS/ALS/spinal muscle atrophy	9037	17,831	3069	3692
Code 74	Parkinson’s disease/extrapyramidal disorder	15,077	14,063	1474	2408
Code 76	Paraplegia, including traumatic and partial	2553	25,125	2455	3220
Code 77	Neurotraumata	2272	8166	1609	1765
Code 78	Other neurological disorders	12,171	15,652	3820	4089
Code 79	Psychomotor retardation/development disorders	117,681	2607	738	832
Code 94	Other collagen disorders	4219	9252	1800	1782
Code 95	Scar tissue	239	8642	1701^ns^	1897
Code 96	Scleroderma	502	16,505	2078	2334
Code 99	Other skin disorders	537	8354	472^ns^	657
PDG-modality 2					
Code 0x	Surgery, musculoskeletal system	2536	15,773	1820	1924
Code 1x	Orthopedic disorders without surgery	27,234	3387	689	858
Code 2x	Surmenage, degenerative disorders, dystrophy	4692	8392	1054	1151
Code 3x	Traumatic disorders excl. surgery/neurotraumata/paraplegia	149	7254	1896	1910
Code 4x	Disorders of heart/veins/lymphatic system incl. cardio surgery	21,578	9146	386	469
Code 5x	Lung disorders	24,769	14,984	2098	2343
Code 6x	Other internal disorders excl. surgery/musculoskeletal system	3733	19,393	4432	4641
Code 7x	Neurological disorders	198,031	7267	1401	1654
Code 9x	Collagen disorders and skin disorders	5494	9840	1699	1709
PDG-modality 3					
Cluster 1	Included diagnoses: 10, 11, 12, 13, 28, 46, 71, 79, 99	175,508	3859	661	779
Cluster 2	Included diagnoses: 0, 39, 54, 69, 70, 74, 77, 94, 95	57,039	13,414	1715	2095
Cluster 3	Included diagnoses: 14, 56, 72, 73, 76, 78, 96	52,770	15,223	2984	3295
Cluster 4	Included diagnoses: 51, 65	1025	35,571	16,340	17,056

^a^ *N* = the number of patients weighted by the duration of enrollment in 2013. For PDG-modality 1 and 2, enrollees may be classified in more than one diagnosis. For PDG-modality 3, enrollees are classified in the cluster containing the applicable diagnosis with the highest residual costs

^b^Residual costs based on the RE-model of 2016 without the PUGs as a risk-adjuster. The subscript ‘ns’ indicates that the residual is not statistically significant different from 0 at a significance level of 1%

^c^Coefficient based on the RE-model of 2016 with the PUGs or with the PUGs replaced by PDG-modality 1, 2 or 3. For all PDG-modalities, enrollees are classified in the applicable diagnosis with the highest residual costs. All coefficients are statistically significant different from 0 a significance level of 1%

In modality 1, residual costs vary from €352 to €22,730. The estimated coefficients are on average approximately €250 higher than the residual costs, which means that these risk-classes take over some of the predictive power of the other risk-adjusters in the model. The variation in coefficients for modality 2 is much smaller as compared to modality 1.

The (residual) costs and thus the coefficients of the clusters of modality 3 sharply increase as the cluster number goes up. An additional analysis shows that the clusters explain 94% of the variance in residual costs among the 27 diagnoses, implying almost no information loss as a result of the clustering. Compared to modality 2, the (residual) cost variation is much better captured, as becomes clear from the variation in coefficients.

In modality 4, over 75% of the patients classified in the first cluster are below age 18, while the 3 other clusters consist for 95% of adult patients (data not shown). This implies that if the RE-model includes modality 1, 3 or 4, the substantial residual-cost differential between children and adults with a physiotherapy diagnosis (i.e. €770 versus €1936) is already accounted for. Within the clusters, the coefficients vary by age (see supplemental digital content 2), but this has limited meaning when the prevalence per risk-class is taken into account.

Finally, the fit statistics in Table 3 imply that while all PDG-modalities (slightly) outperform the PUGs in terms of predictive power, modality 3 is preferred over the other modalities in view of its equal or better fit and the lowest number of risk-classes.

**Table 3:** Fit statistics on the individual-level for five versions of the Dutch RE-model of 2016

Model	*R* ^2^ × 100%^a^	CPM × 100%^b^	MAPE^c^
RE-model 2016 without PUGs	25.33	27.19	1976.54
RE-model 2016 with PUGs	25.36	27.28	1973.97
RE-model 2016 without PUGs + PDG-modality 1	25.45	27.32	1972.83
RE-model 2016 without PUGs + PDG-modality 2	25.41	27.28	1973.95
RE-model 2016 without PUGs + PDG-modality 3	25.45	27.33	1972.78
RE-model 2016 without PUGs + PDG-modality 4	25.45	27.33	1972.69

^a^ *R* ^2^ = proportion explained variance = 1−(sum of squared residuals/total sum of squared differences)

^b^ CPM = Cummings’ prediction measure = 1−(sum of absolute residuals/total sum of absolute differences)

^c^ MAPE = mean absolute prediction error = sum of absolute residuals divided by the weighted number of patients

## Discussion and conclusion

Although RE-models in competitive health insurance markets have become increasingly sophisticated, they often still result in substantial undercompensations for specific groups of people in poor health. A potentially viable option to further improve RE-models is to make use of information on (diagnoses of) treatments in primary care instead of only hospital care (as is common in current RE-models [3, 4, 7, 8]). Hence, a new risk-adjuster based on the prior use of physiotherapy (‘physiotherapy utilization groups’, PUGs) was introduced in the Dutch RE-model of 2016. However, this risk-adjuster has clear disadvantages since it is based on prior costs and does not take the severity of the underlying diagnosis into account.

Using physiotherapy claims data of 2012 and administrative data on costs and risk characteristics of 2013 for almost the entire Dutch population, this paper investigated the possibilities of improving RE using information on (use of) physiotherapy by (1) evaluating the current cost-based PUGs and (2) assessing the effects of replacing this risk-adjuster by 4 different modalities of an alternative risk-adjuster based on 27 physiotherapy diagnoses related to specific chronic conditions (‘physiotherapy diagnosis groups’, PDGs).

Although adding the PUGs removes the average undercompensation of €919 for the subgroup of enrollees with physiotherapy costs in the prior year and (slightly) improves the model’s predictive power, all 4 PDG-modalities have clear advantages over the PUGs in terms of validity, stability, measurability, incentives for efficiency, predictive power, and simplicity/transparency.

The PDG-modalities are *more valid* indicators of health than the PUGs because (1) they are based on diagnoses instead of on costs, (2) only diagnoses pertaining to chronic conditions are included instead of also temporary health problems, (3) they take the severity of the underlying diagnoses into account, and (4) all ages are included instead of adults only.

In addition, insurers’ *efficiency incentives* are not reduced since the PDG-modalities are not based on prior costs. On the other hand, an inherent disadvantage of using diagnosis-based risk-adjusters is that it may provide incentives for insurers to promote classifying patients into more ‘severe’ diagnoses-categories than is medically necessary because these diagnoses are associated with higher payment. However, while in general such upcoding practices can be a significant problem in diagnosis-based payment schemes, it is unlikely to be very relevant in the context of this study. First, physiotherapy treatment for adults for the conditions related to the 89 analyzed diagnoses is not covered under the Dutch basic health insurance until the 21st treatment. Although for children coverage does start at the first treatment, the estimated coefficients for the diagnoses that are most relevant for this group are relatively low (i.e. the average coefficient for these diagnoses is lower than that of the current PUGs), mitigating possible upcoding incentives. More importantly, the Dutch physiotherapy diagnosis classification system contains only 89 different and clearly distinct (pathology) categories. Of these, only 27 are relevant for the PDG-modalities, which all pertain to serious ‘permanent’ *chronic* conditions for which upcoding does not seem to be a real issue. Still, although we thus believe there to be limited potential for upcoding in this context, as with any diagnosis-based payment scheme, monitoring changes in diagnosis prevalence over time as well as periodic validation of set diagnoses (e.g. by contrasting diagnosis prevalence patterns with utilization patterns of related types of care) is advisable.

Regarding *measurability*, the advantage of the PDG-modalities is that no arbitrary decisions are required for determining when someone will be classified in the risk-adjuster or not. In the current RE-model, enrollees are classified in a PUG > 0 if their physiotherapy costs in the prior year are in the top-2%. Although this threshold was introduced to eliminate the impact of administrative (data) flaws and to mitigate the reduction in efficiency incentives, it was arbitrarily determined. On the other hand, in constructing the PDG-modalities the enrollees of three insurers were necessarily dropped because of lacking or inadequate claims. However, this problem appears to have been solved in the 2013-claims data.

Regarding *stability*, this paper has shown that in terms of the number of patients classified, the PDGs are more stable at the insurer-level than the PUGs. In addition, the prevalence of the 27 diagnoses varies only marginally over time. Although our data did not permit analysis of the stability of coefficients, findings from the recent annual update of the RE-model confirm that the (pattern in the) coefficients of PDG-modality 3 (i.e. the 27 diagnoses clustered in 4 risk-classes based on residual costs) are indeed stable [18]: the weighted average absolute change in estimated coefficients from 2013 to 2014 equals €2.

Relative to the PUGs, all PDG-modalities (slightly) improve the model’s *predictive power*. In turn, modality 3 outperforms the other modalities given its low number of risk-classes and comparable or better predictive power.

Finally, all PDG-modalities are arguably more *transparent* than the PUGs because (1) they show considerably less overlap with the ‘morbidity-based’ adjusters already in the model, and (2) ‘diagnoses’ are more salient and easier to interpret than ‘costs’. Of the 4 modalities, modality 3 may be viewed as being the simplest because it consists of only 4 risk-classes. However, as a result of the clustering method these risk-classes have lost their (pathology) label and thus are not clinically recognizable, reducing the transparency. On the other hand, modality 3 is based on the same clustering method as the current DCGs [14], enhancing model consistency.

Our findings must be viewed in the light of three limitations. First, about 1 million enrollees of three insurers were necessarily dropped from the analysis due to inadequate information on physiotherapy claims of 2012. Nonetheless, analysis was possible for 94% of the Dutch population. Second, our data did not permit analysis of the stability of the coefficients of the PDG-modalities. However, findings of follow-up research indicate that the coefficients of modality 3 are indeed stable [18]. Finally, both the 2012 and the 2013 data contain claims that are actually not covered under the basic health insurance. However, these claims (which were excluded from the analysis) comprise less than 2% of the total number of claims in both years.

In conclusion, adding information on physiotherapy (diagnoses) can further improve the performance of already sophisticated RE-models. Of the different physiotherapy-based risk-adjuster modalities analyzed in this paper, the modality based on clustered physiotherapy diagnoses based on residual costs is the preferred modality. Compared to a risk-adjuster based on prior physiotherapy costs, such a diagnosis-based risk-adjuster is more valid, more stable, more easily measured, more transparent, has more predictive power, and does not compromise insurers’ incentives for promoting efficiency of care.

## Electronic supplementary material

Below is the link to the electronic supplementary material.

Supplementary material 1 (PDF 714 kb)
