# Supplementary material for: Improving risk equalization using information on physiotherapy diagnoses
Source: Eur J Health Econ. 2017 Feb 9;19(2):203–11. doi: 10.1007/s10198-017-0874-x (PMC5813071; doi:10.1007/s10198-017-0874-x)

Improving risk equalization using information on physiotherapy diagnoses

European Journal of Health Economics

Frank Eijkenaar, PhD (corresponding author)

René C.J.A. van Vliet, PhD

Institute of Health Policy and Management, Erasmus University Rotterdam

Burgemeester Oudlaan 50, 3000 DR Rotterdam, The Netherlands

T: +31 10 408 9183

F: +31 10 408 9094

E: eijkenaar@bmg.eur.nl

E: r.vanvliet@bmg.eur.nl

SDC 1. Chronic conditions covered by Dutch basic health insurance in 2016: all treatments for children (age ≤17) and from the 21st treatment for adults (age ≥18). *

Condition	Limitation of coverage
Disorders of the nervous system	
1. Cerebrovascular accident	-
2. Bone marrow disorder	-
3. Multiple sclerosis	-
4. Peripheral nerve disorder with motoric outfall	-
5. Extrapyramidal disorder	-
6. Motoric retardation or development disorder	Ages 0-16 only
7. Congenital disorder of the central nervous system	-
8. Cerebellar disorder	-
9. Symptoms of outfall resulting from tumor in brain or bone marrow, or from brain damage	-
10. Radicular syndrome with motoric outfall	Maximum treatment duration: 3 months
11. Muscle disease	-
12. Myasthenia gravis	-
Disorders of the musculoskeletal system:	
1. Congenital disorder	-
2. Progressive scoliosis	-
3. Juvenile osteochondrosis	Ages 0-21 only
4. Reflex dystrophy	-
5. Fracture resulting from Kahler's disease, bone metastasis, or Paget's disease	-
6. Frozen shoulder	Maximum treatment duration: 12 months
7. Hyperostotic spondylosis (Forestier's disease)	-
8. Collagen disorders	-
9. Status after amputation	-
10. Whiplash	Maximum treatment duration: 9 months
11. Postpartum instability of the pelvis	Maximum treatment duration: 3 months
12. Fractures treated conservatively	Maximum treatment duration: 6 months
Other disorders:	
1. Chronic obstructive pulmonary disease	Stage II or higher of GOLD classification
2. Congenital disorder of the respiratory system	-
3. Lymph edema	-
4. Scar tissue of the skin, possibly after trauma	-
5. Status after admission in a hospital/nursing home/revalidation	Maximum treatment duration 12 months
institute, or after outpatient treatment in revalidation institute	-
6. Claudicatio intermittens (vascular), Fontaine stage 2/3	Maximum treatment duration 12 months
7. Soft tissue tumors	Maximum treatment duration 24 months
8. Diffuse interstitial lung disorder in case of ventilator limitation or diffusion disorder	-

* Source: http://wetten.overheid.nl/BWBR0018492/2016-01-01#Bijlage1.

SDC 2. Estimated coefficients (in €)

Risk classes	RE-model 2016 - PUGs	RE-model 2016 + PUGs	Re-model 2016 - PUGs + PDG-modality 1	Re-model 2016 - PUGs + PDG-modality 2	Re-model 2016 - PUGs + PDG-modality 3	Re-model 2016 - PUGS + PDG-modality 4
Male, 0	4989	4999	4970	4943	4971	4983
Male, 1-4	1847	1860	1809	1766	1811	1823
Male, 5-9	1659	1672	1614	1565	1617	1628
Male, 10-14	1539	1551	1522	1505	1522	1533
Male, 15-17	1561	1573	1561	1561	1558	1569
Male, 18-24	1402	1407	1413	1417	1412	1398
Male, 25-29	1381	1384	1393	1398	1393	1378
Male, 30-34	1391	1394	1403	1409	1403	1387
Male, 35-39	1480	1484	1493	1500	1493	1477
Male, 40-44	1569	1573	1584	1592	1584	1568
Male, 45-49	1710	1713	1725	1736	1725	1709
Male, 50-54	1877	1880	1895	1908	1895	1879
Male, 55-59	2196	2195	2212	2229	2212	2197
Male, 60-64	2445	2445	2465	2484	2464	2449
Male, 65-69	2968	2964	2948	2933	2951	2991
Male, 70-74	3367	3363	3345	3329	3348	3387
Male, 75-79	3857	3854	3831	3816	3834	3872
Male, 80-84	4521	4522	4496	4484	4497	4536
Male, 85-89	5514	5522	5500	5491	5501	5540
Male, 90+	7683	7690	7677	7668	7677	7716
Female, 0	4404	4415	4395	4377	4395	4407
Female, 1-4	1600	1613	1573	1541	1575	1586
Female, 5-9	1528	1541	1515	1496	1516	1527
Female, 10-14	1511	1523	1501	1493	1500	1511
Female, 15-17	1695	1708	1692	1691	1688	1698
Female, 18-24	1719	1725	1730	1735	1729	1714
Female, 25-29	2180	2186	2195	2203	2195	2179
Female, 30-34	2338	2344	2356	2365	2355	2340
Female, 35-39	2019	2023	2036	2046	2035	2020
Female, 40-44	1761	1761	1776	1787	1775	1761
Female, 45-49	1828	1823	1839	1853	1838	1825
Female, 50-54	1938	1929	1948	1964	1947	1935
Female, 55-59	2084	2069	2090	2110	2089	2078
Female, 60-64	2253	2236	2260	2281	2259	2248
Female, 65-69	2625	2602	2598	2583	2600	2636
Female, 70-74	2932	2906	2907	2891	2909	2945
Female, 75-79	3510	3484	3485	3470	3486	3523
Female, 80-84	4552	4530	4531	4519	4531	4567
Female, 85-89	5989	5972	5967	5960	5966	6001
Female, 90+	8102	8090	8091	8087	8088	8125
No PCG	-306	-306	-313	-324	-312	-311
Glaucoma	196	204	226	247	224	226
Thyroid disorders	98	102	140	175	136	133
Psychosis/Alzheimer/additction	293	329	382	410	379	367
Depression	318	323	363	412	357	348
Chronic pain excl. opioids	999	977	1023	1051	1022	1021
Neuropathic pain complex	2158	2108	2061	2127	2072	2079
High cholesterol	90	92	122	169	114	114
Diabetes II, no hypertension	639	647	688	734	684	681
COPD/severe asthma	2037	1993	1937	1917	1935	1932
Asthma	596	600	631	677	626	620
Diabetes II plus hypertension	938	951	988	1022	982	983
Epilepsy	1369	1359	1333	1380	1326	1324
Crohn’s disease	1121	1143	1187	1222	1184	1178
Heart conditions	2438	2459	2477	2482	2473	2473
Auto-immune disease (add-on)	12936	12982	13027	13032	13026	13021
Rheumatoid arthritis	994	1003	1018	1019	1014	1015
Parkinson’s disease	3578	3208	2527	2895	2679	2637
Diabetes I	2097	2143	2203	2225	2198	2196
Transplantations	233	281	316	340	309	308

Risk classes	RE-model 2016 - PUGs	RE-model 2016 + PUGs	Re-model 2016 - PUGs + PDG-modality 1	Re-model 2016 - PUGs + PDG-modality 2	Re-model 2016 - PUGs + PDG-modality 3	Re-model 2016 - PUGS + PDG-modality 4
CF/pancreas-enzymes	3682	3741	3128	3664	2853	2853
Multiple sclerosis	-1072	-1166	-1749	-1189	-1636	-1427
Other brain/spinal cord disorder	4948	4617	3843	4395	3894	4031
Cancer	1450	1471	1512	1527	1507	1502
Hormone sensitive tumors	905	829	908	881	879	878
HIV/AIDS	1045	1237	1404	1359	1407	1403
Kidney conditions	6600	6623	6737	6694	6743	6744
Psoriasis	503	522	546	582	542	537
Pulmonal arterial hypertension	17520	17583	17692	17636	17636	17647
Cancer (add-on)	8820	8795	8927	8851	8902	8912
Growth disorder (add-on)	-920	-717	-592	-654	-569	-582
No DCG	-259	-257	-260	-264	-260	-260
DCG 1	567	564	613	645	606	604
DCG 2	521	531	574	613	569	566
DCG 3	1163	1123	1217	1251	1214	1213
DCG 4	2079	2055	2059	2080	2058	2057
DCG 5	2327	2315	2327	2368	2317	2309
DCG 6	3251	3192	3211	3265	3192	3193
DCG 7	5312	5286	5228	5307	5241	5257
DCG 8	6081	6075	5923	5959	5948	5960
DCG 9	10365	10364	10305	10316	10322	10318
DCG 10	11011	10910	10625	10876	10647	10683
DCG 11	16580	16573	16435	16543	16473	16470
DCG 12	21839	21813	21817	21807	21808	21814
DCG 13	36134	35997	35326	35848	35475	35551
DCG 14	50081	50145	50054	50278	50128	50117
DCG 15	65903	66022	66210	66132	66221	66214
No DMECG	-28	-28	-28	-28	-28	-28
Insulin drip-pumps	-280	-206	-135	-134	-137	-137
Catheters/urine-containers	3400	3376	3317	3406	3321	3324
Stoma	3450	3505	3542	3555	3546	3551
Tracheo-stoma	12740	12794	12797	12770	12808	12801
Reference 18- en 65+	0	0	0	0	0	0
Full disability benefits 18-34	2204	2141	1979	2126	1983	2051
Full disability benefits 35-44	1852	1767	1597	1741	1609	1685
Full disability benefits 45-54	1777	1695	1540	1652	1539	1618
Full disability benefits 55-64	1041	964	853	912	848	910
Part. disability benefits 18-34	526	513	493	522	497	508
Part. disability benefits 35-44	648	627	612	643	617	632
Part. disability benefits 45-54	586	565	550	576	554	570
Part. disability benefits 55-64	477	459	446	463	448	462
Social security benefits 18-34	238	239	240	242	240	239
Social security benefits 35-44	254	255	256	261	256	256
Social security benefits 45-54	336	341	342	346	342	342
Social security benefits 55-64	316	323	325	326	325	325
Students 18-34	-227	-228	-226	-227	-225	-226
Self-employed 18-34	-118	-116	-117	-119	-117	-118
Self-employed 35-44	-171	-169	-169	-173	-170	-171
Self-employed 45-54	-246	-241	-243	-247	-243	-245
Self-employed 55-64	-328	-322	-322	-328	-323	-326
Higher-educated 18-34	-38	-38	-36	-38	-36	-37
Reference 18-34	23	25	25	24	25	24
Reference 35-44	-41	-40	-38	-41	-39	-40
Reference 45-54	-60	-58	-56	-59	-56	-58
Reference 55-64	-91	-87	-83	-87	-84	-87
Region 1	81	84	85	85	85	85
Region 2	43	44	45	44	45	45
Region 3	14	14	14	14	14	14
Region 4	8	8	8	8	8	8
Region 5	-6	-7	-7	-6	-7	-7
Region 6	-9	-10	-10	-10	-10	-10
Region 7	-23	-24	-25	-25	-25	-25

Risk classes	RE-model 2016 - PUGs	RE-model 2016 + PUGs	Re-model 2016 - PUGs + PDG-modality 1	Re-model 2016 - PUGs + PDG-modality 2	Re-model 2016 - PUGs + PDG-modality 3	Re-model 2016 - PUGS + PDG-modality 4
Region 8	-21	-22	-22	-21	-22	-22
Region 9	-44	-45	-45	-45	-45	-44
Region 10	-45	-45	-46	-46	-46	-46
Address>15 persons 18-	203	205	207	203	207	207
Address>15 persons stay 18-64	-249	-234	-235	-243	-234	-237
Address>15 persons stay 65+	-2120	-2128	-2168	-2149	-2173	-2185
Address>15 persons new 18-64	699	708	703	702	703	704
Address>15 persons new 65+	3251	3247	3211	3221	3211	3203
Income deciles 1-2 18-	68	67	69	71	69	69
Income deciles 1-2 18-64	46	48	47	46	47	47
Income deciles 1-2 65+	856	859	858	855	858	857
Income deciles 3-4 18-	18	18	17	15	17	17
Income deciles 3-4 18-64	21	21	21	22	21	21
Income deciles 3-4 65+	61	60	61	59	61	61
Income deciles 5-7 18-	-22	-22	-24	-26	-24	-24
Income deciles 5-7 18-64	7	6	7	7	7	7
Income deciles 5-7 65+	-204	-204	-202	-203	-202	-201
Income deciles 8-10 18-	-37	-37	-35	-33	-35	-35
Income deciles 8-10 18-64	-51	-52	-52	-53	-52	-52
Income deciles 8-10 65+	-318	-317	-315	-314	-315	-315
No MYHCG	-309	-301	-297	-300	-297	-297
2x costs in top-10%	2721	2610	2650	2679	2646	2643
3x costs in top-15%	2545	2458	2425	2461	2425	2424
3x costs in top-10%	4317	4182	4103	4151	4104	4097
3x costs in top-7%	6951	6769	6641	6707	6643	6632
3x costs in top 4%	12015	11797	11608	11700	11608	11600
3x costs in top 1,5%	31120	30900	30508	30729	30488	30494
Healthy 65-	0	2	16	34	14	12
Healthy 65+	-226	-226	-191	-159	-195	-186
Unhealthy 65-	-2	-10	-73	-155	-66	-53
Unhealthy 65+	148	144	124	103	127	121
PUG0 (no physio-costs t-1)		-21				
PUG1 (yes physio-costs t-1)		1008				
No physio-diagnosis			-30			
00			1971			
10			423			
11			435			
12			1334			
13			567			
14			6125			
28			1173			
39			1889			
46			471			
51			12049			
54			2117			
56			3534			
65			23014			
69			2058			
70			1918			
71			1281			
72			2773			
73			3692			
74			2408			
76			3220			
77			1765			
78			4089			
79			832			
94			1782			
95			1897			
96			2334			
99			657			
No physio-diagnosis				-30		

Risk classes	RE-model 2016 - PUGs	RE-model 2016 + PUGs	Re-model 2016 - PUGs + PDG-modality 1	Re-model 2016 - PUGs + PDG-modality 2	Re-model 2016 - PUGs + PDG-modality 3	Re-model 2016 - PUGS + PDG-modality 4
Main pathology category 0				1924		
Main pathology category 1				858		
Main pathology category 2				1151		
Main pathology category 3				1910		
Main pathology category 4				469		
Main pathology category 5				2343		
Main pathology category 6				4641		
Main pathology category 7				1654		
Main pathology category 9				1709		
No physio-diagnosis					-29	
Cluster 1					779	
Cluster 2					2095	
Cluster 3					3295	
Cluster 4					17056	
No physio-diagnosis 18-						-40
No physio-diagnosis 18-64						-12
No physio-diagnosis 65+						-77
Cluster 1 18-						749
Cluster 1 18-64						405
Cluster 1 65+						1451
Cluster 2 18-						1725
Cluster 2 18-64						1844
Cluster 2 65+						2242
Cluster 3 18-						5104
Cluster 3 18-64						2503
Cluster 3 65+						3775
Cluster 4 18-						16415
Cluster 4 18-64						17629
Cluster 4 65+						17151

SDC 3. Descriptive statistics for the 89 original diagnoses (in €) *

Code	Description †	N (weighted)	Mean costs	Mean residual costs ‡	Std. dev. of mean residual costs ‡
00	Amputation	2536	15603	1737	17363
01	*Joints, excl. vertebrae column, meniscectomy, synovectomy*	65795	5613	-238	9865
02	*Bones, excl. vertebrae column*	17252	4917	320	8575
03	*Meniscectomy, synovectomy*	11240	2834	252	5366
04	*Tendon, muscle, vinculum*	20153	2708	254	5386
05	*Vertebrae column*	11740	5478	136 [ns]	8769
06	Removed osteosynthesis material	428	4414	701 [ns]	7862
08	*Postoperative contracture/atrophy*	1156	6747	1022 [ns]	21843
09	Other surgery of musculoskeletal system (incl. tumors)	2869	7945	553 [ns]	15854
10	Aseptic bone necrosis	375	1377	354 [ns]	2317
11	Disorders of the vertebral column / pelvis	19344	2333	355	5470
12	Congenital disorders of the skeleton	5896	5250	1235	11540
13	Ossification disorder	1107	4311	473	6651
14	Inflammation of/tumors in the skeleton	676	16476	5948	25415
15	Pseudo-arthrosis/epiphysiolysis/apophysis	160	1559	625 [ns]	4522
16	*Stand disorders extremities*	3161	1274	184	2810
17	Disorders of joints, excluding vertebrae column pelvis	1640	1265	256	2365
18	Other orthopedic conditions without surgery	4963	1218	-3	2778
19	Dupuytren contracture	7	5515	2773 [ns]	8424
20	Epicondylitis, tendinitis, tendovaginitis	10804	1064	235	2773
21	*Bursitis (non-traumatic)/capsulitis*	12288	4446	468	10233
22	Chondropathy/arthropathy, meniscal lesion	22977	1227	314	4593
23	Arthrosis	322	2928	57	4855
24	Osteoporosis	213	11005	1637 [ns]	14581
25	Syndrome of Costen	234	1337	319	2761
26	Muscle, tendon, and fascia conditions	98174	1142	245	3765
27	Discusdegeneration, coccydynia/HNP	1281	1302	264	2347
28	Sudeck' a(dys)trophy	4692	8351	1043	9839
31	Jointcontusion/-distorsion	23850	1207	326	3453
32	*Luxation (sub-)*	9593	1741	214	3949
33	*Rupture of muscle or tendon, hematoma*	6088	1583	313	4749
34	Hydrops, haemarthros, traumatic edema	579	1632	395	4475
35	Myositis ossifica /adhaesions/traumatic bursitis	192	1418	305 [ns]	3313
36	*Fractures*	18267	4613	523	8919
38	*Whiplash injury (neck trauma)*	1093	2772	738	6171
39	Status after burn wounds	149	7063	1732	8943
40	Heart conditions (excluding codes 41 to 45)	166	11232	2138 [ns]	17469
41	Acute myocardial infarcton (AMI)	66	10299	1377 [ns]	17035
42	Status after coronary artery bypass grafting	87	9532	-469 [ns]	9142
43	Status after percutaneous transluminal coronary angioplasty	35	7649	-1633 [ns]	7697
44	Status after heart valve operation	68	10204	584 [ns]	11692
45	Status after surgically corrected congenital disorder	75	20333	11414	31962
46	Disorders of the lymphatic system / edema	21578	9120	399	13198
47	Ulcus/decubitus/necrosis	6	18335	3924 [ns]	10987
48	*General vascular disease, circulation disorders*	7844	9518	132 [ns]	14854
50	Chronic aspecific respiratory condition	1211	2911	556	6352
51	Congenital disorders of respiratory system	566	35374	11253	41526
52	Pneumothorax/pulmonary edema	22	2769	-1877 [ns]	8037
53	Respiratory infections	212	5155	2052	10116
54	Chronic obstructive pulmonary disease	23309	14470	1840	18143
55	Emphysema	70	15214	1423 [ns]	13692
56	Interstitial lung disorder including sarcoïdosis	920	14792	3767	26538

Code	Description †	N (weighted)	Mean costs	Mean residual costs ‡	Std. dev. of mean residual costs ‡
60	Diabetes mellitus	57	9719	2652 ^{ns}	12175
61	Immunity disorders	53	11978	5367	19685
62	Spastic colon	168	2412	720	4955
64	Adipositas	2736	1584	594	9075
65	Other hereditary disorders	460	35808	22767	99176
68	*Surgery excl. muskoloskeletal system and cardiology*	3862	12811	97 ^{ns}	18929
69	Tumors without surgery	3273	17060	1915	26903
70	Peripheral nerve disorder	6404	9791	1819	13577
71	Cerebellar disorders	6755	11562	1068	16427
72	Cerebrovascular accident / central paresis	27050	13049	2566	15551
73	MS / ALS / spinal muscle atrophy	9037	17831	3069	22254
74	Parkinson’s disease / extrapyramidal disorder	15077	14063	1474	15792
75	*HNP with radicular syndrome*	1176	4124	424 ^{ns}	7090
76	Paraplegia, including traumatic and partial	2553	25125	2455	28557
77	Neurotraumata	2272	8166	1609	11265
78	Other neurological disorders	12171	15652	3820	33724
79	Psychomotor retardation/development disorders	117686	2607	738	10591
80	Symptomatology without assignably pathology)	25060	1401	273	7511
81	Psychosomatic conditions	2998	1506	433	4153
82	Hyperventilation without pulmonary pathology	3207	1458	441	4330
83	Proctology	52	1669	253 ^{ns}	2449
84	Stomach, intestines, liver	462	1964	681	3220
85	Sexology	109	1606	144 ^{ns}	2036
86	Urine incontinence	17622	3895	214	8704
87	Fecal incontinence	681	2542	793	6998
88	Urology	1475	1446	243	2741
89	*Gynecology*	238	2938	380 ^{ns}	5711
90	(Chronic) rheumatoid arthritis	548	10619	928 ^{ns}	11060
91	Juvenile rheumatoid arthritis	235	7893	2020	7618
92	(Poly)arthritis	324	11773	3171 ^{ns}	25420
93	Ankylosing spondylitis (Bechterew’s disease)/ankylosis	213	6884	-521 ^{ns}	5487
94	Other collagen disorders	4219	9252	1800	17700
95	Scar tissue	239	8642	1701 ^{ns}	19527
96	Scleroderma	502	16505	2078	24328
97	Psoriasis	159	3531	1190 ^{ns}	5888
98	Hyperhidrosis	25	2138	-214 ^{ns}	3023
99	Other skin disorders	537	8354	472 ^{ns}	10146

* N = the number of patients weighted by the duration of enrollment in 2013. Enrollees may have more than one diagnosis.

† The underlined diagnoses are the 27 diagnoses included in this study, i.e. they are covered for adults for an unlimited treatment duration (see SDC1 above). The diagnoses in *italics* are also on the chronic list by only covered with limited treatment duration. The remaining diagnoses are only covered for individuals up to age 18, except for diagnosis 86, which is only relevant for adults.

‡ Residual costs based on the RE-model of 2016 without the PUGs as a risk-adjuster. The subscript ‘ns’ indicates that the residual is not statistically significant different from 0 at a significance level of 1%.

SDC 4. Validity checks

Figure A.4.1 shows the total physiotherapy costs in the 2012 claims data as a percentage of the total physiotherapy costs in the 2012 costs data. The overall agreement is high (96%), although for 7 insurers agreement is below 90%. In 6 cases, these are (very) small, non-risk bearing organizations with a mandate to run the basic health insurance on behalf of 1 of the 23 risk-bearing health insurers. The exception is (risk-bearing) insurer 16, which submitted virtually no physiotherapy claims for the year 2012.

Figure A4.1. Physiotherapy costs in the 2012 claims data as a percentage of total physiotherapy costs in the 2012-costs data, per insurer

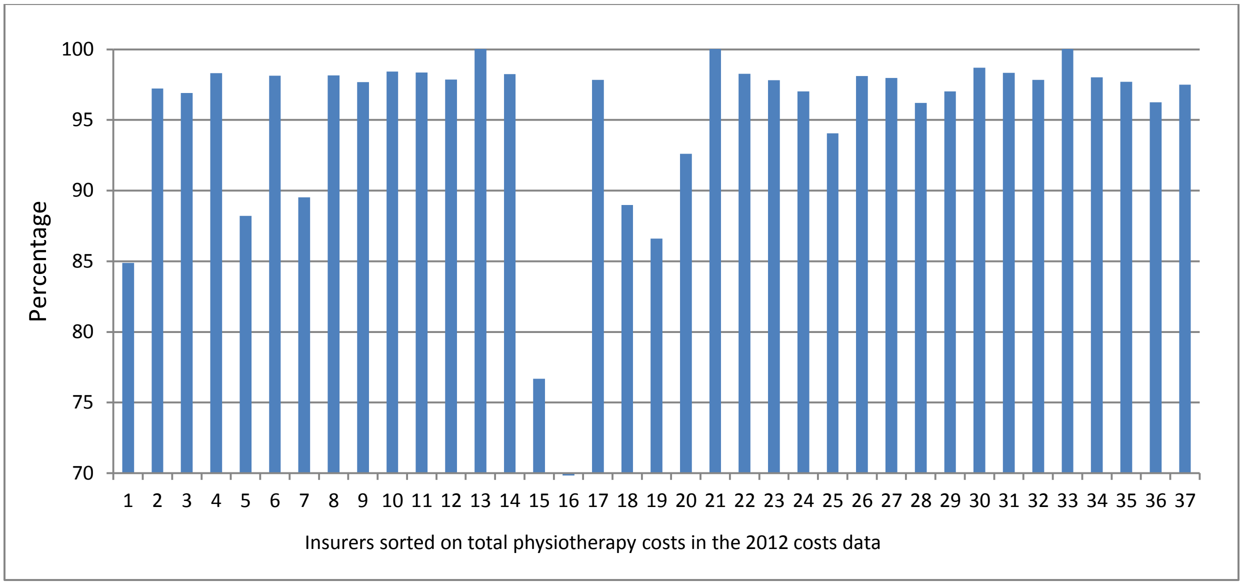

As shown in Figure A.4.2, the individual-level correlation between the total physiotherapy costs in the 2012 claims data and the total physiotherapy costs in the 2012 costs data is typically above 0.97.

Figure A4.2. Individual-level correlation per insurer between total physiotherapy costs in the 2012 claims data and total physiotherapy costs in the 2012 costs data

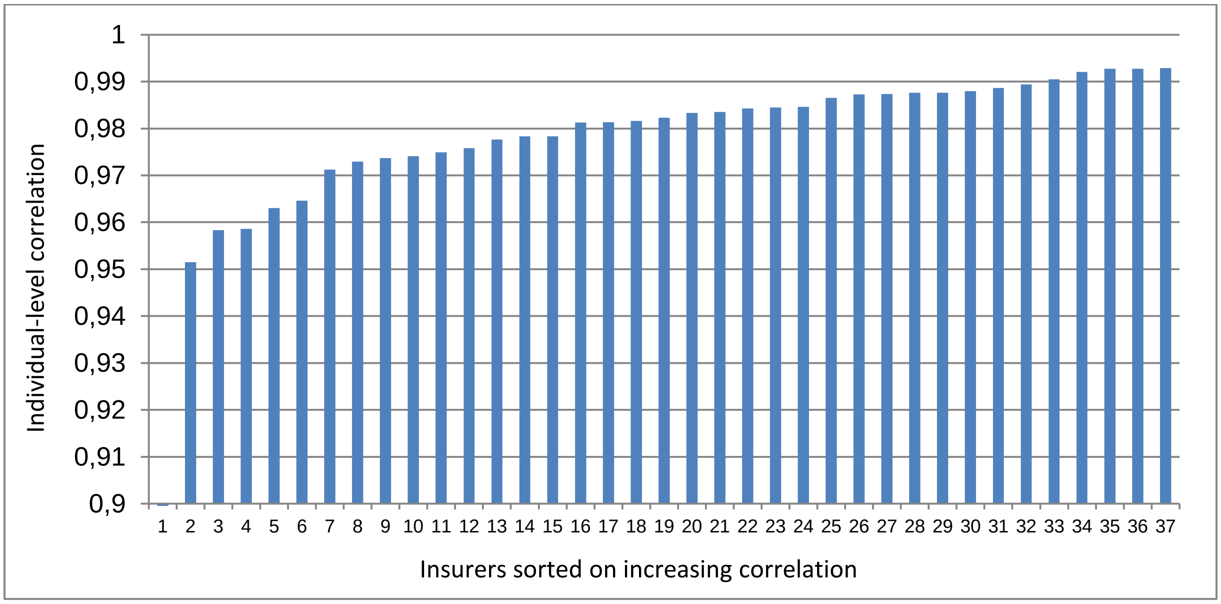

Figure A.4.3 compares the 2012 claims data with the 2012 costs data regarding the mean physiotherapy costs per age-gender group distinguished in the Dutch RE-model. Both data sources show similar costs (patterns). The drop from the age group 15-17 to age group 18-24 is a direct result of the more limited basic health insurance coverage for physiotherapy for adults as compared to children.

Figure A.4.3. Mean physiotherapy costs in 2012 claims data (transparent bars) and 2012 costs data (solid bars) per age-gender group in the Dutch RE-model

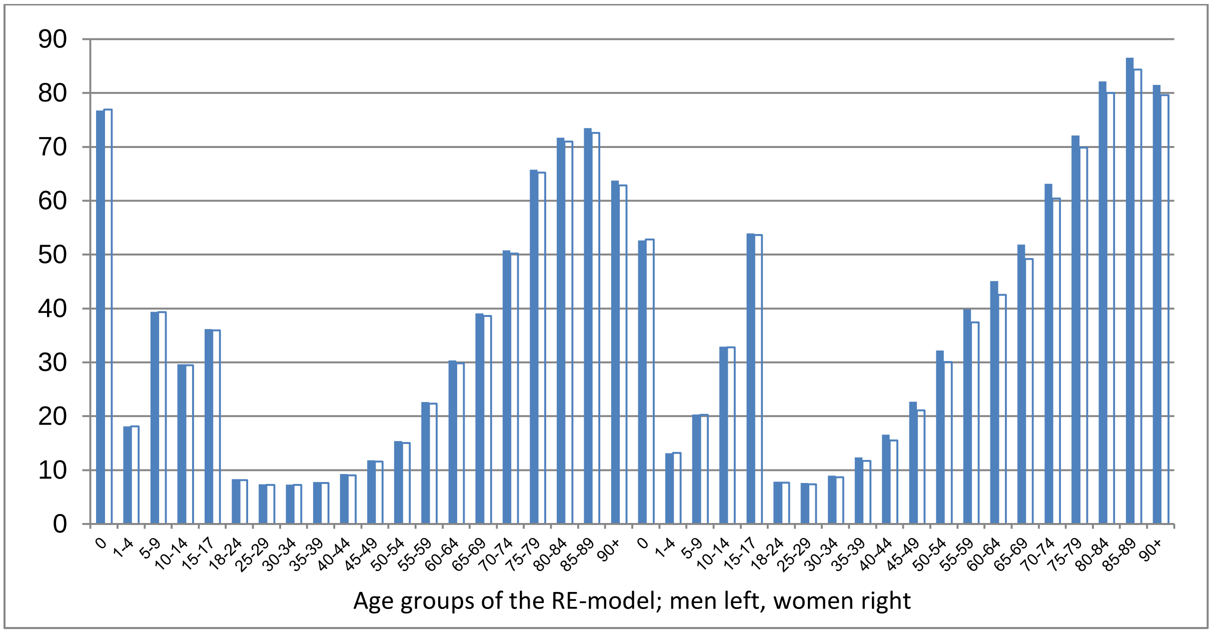

Figures A.4.4 to A.4.6 contain the same comparisons as the 3 figures above, but then for 2013. The 2013 claims data contain approximately 13.7 million claims (2012: 14.4 million) submitted for approximately 673,000 patients (2012: 700,000), and total costs of €426 million (2012: €441 million).

Figure A.4.4. Total physiotherapy costs in the 2013 claims data as a percentage of total physiotherapy costs in the 2013-costs data, per insurer

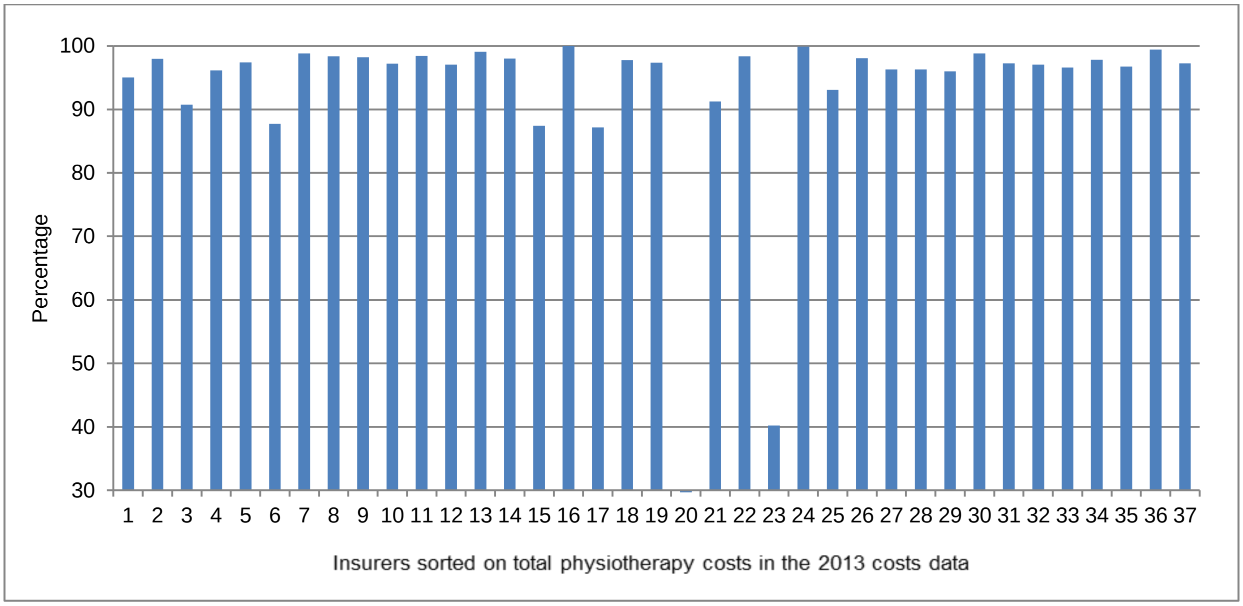

Figure A.4.5. Individual-level correlation per insurer between total physiotherapy costs in the 2013 claims data and the total physiotherapy costs in the 2013 costs data

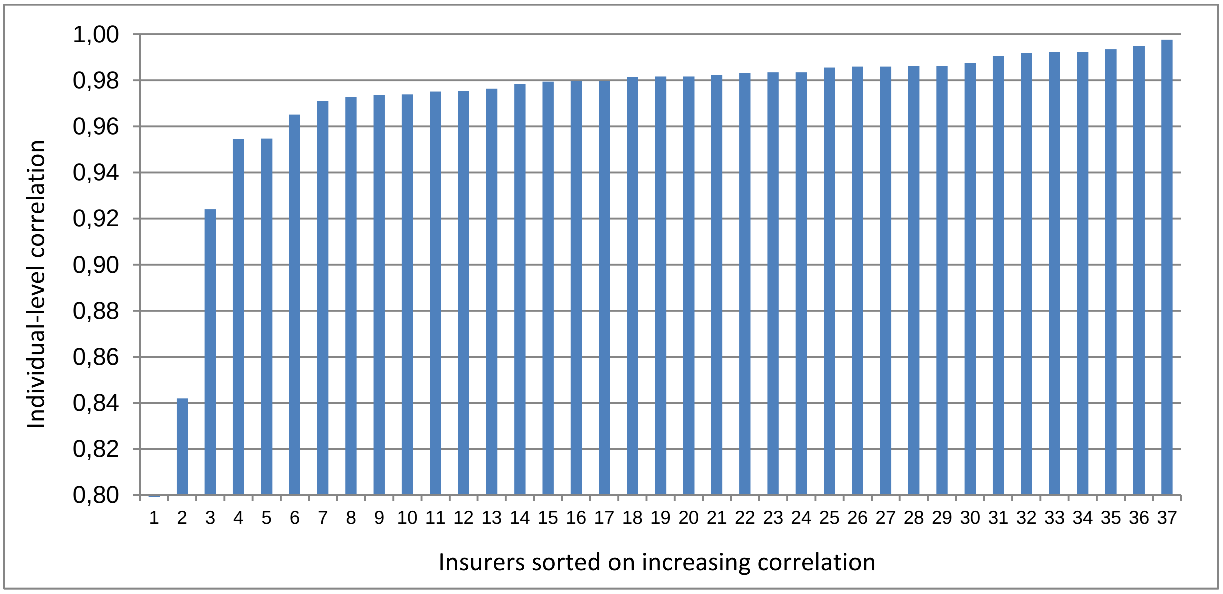

Figure A.4.6. Mean physiotherapy costs in 2013 claims data (transparent bars) and 2013 costs data (solid bars) per age-gender group in the Dutch RE-model

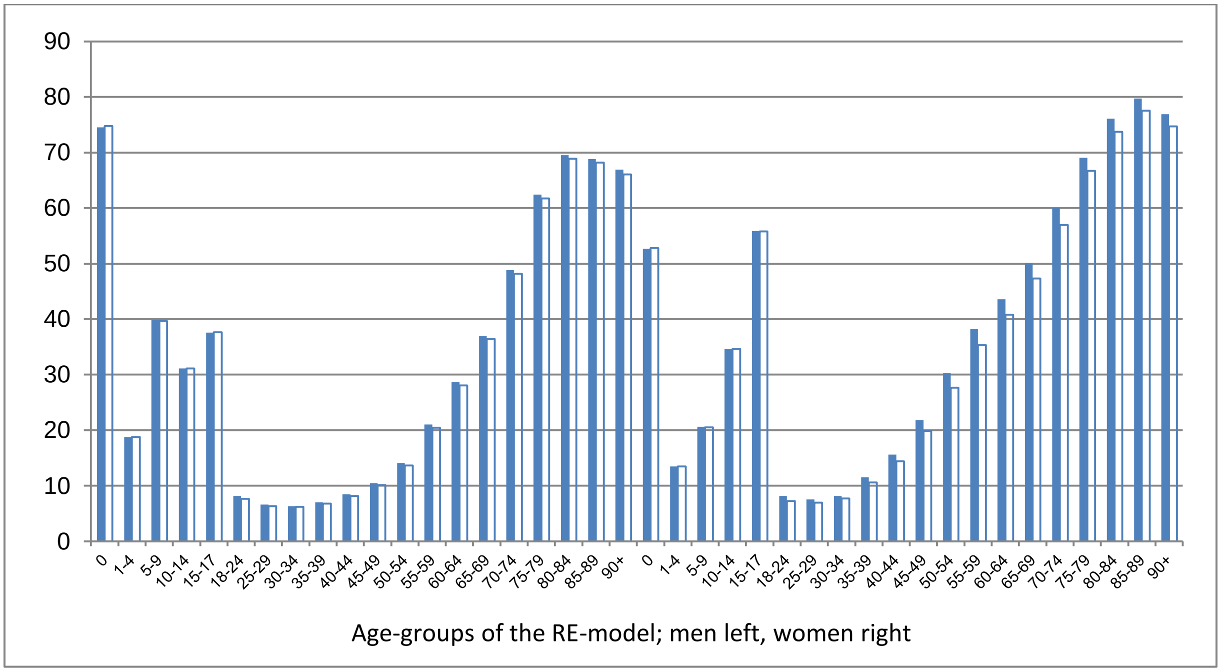

SDC 5. Overlap with current morbidity-based risk-adjusters

As shown in table A.5.1, approximately 85% of the approximately 142,000 individuals age 0-17 with a physiotherapy diagnosis (who are not classified in the current PUGs because the PUGs only apply to adults) are not classified in 1 of the morbidity-based risk adjusters. Apparently, these are children with a chronic condition that is not (yet) severe enough to be classified in a PCG, DCG, DMECG or MYHCG.

Overall, the number of patients classified in 1 of the 4 morbidity-based risk adjusters plus either 1 of the physiotherapy-based risk adjusters increases by approximately 56,200 (i.e. 63,600 less adults due to the restriction to 'permanent' chronic conditions, but 119,800 children extra), despite the fact that the overall prevalence of the 27 diagnoses is about 26,000 patient-years lower than the prevalence of the current PUGs (see the bottom row).

Table A.5.1. Overlap between current morbidity-based risk-adjusters and two physiotherapy-based risk adjusters

Classified in a PCG, DCG, DMECG, and/or MYHCG	Age category	Classified in PUG>0 (i.e. yes physiotherapy costs)	Classified in 1 of the 27 selected physiotherapy diagnoses
No	0-17	0	119,842
	18+	81,818	18,202
Yes	0-17	0	21,983
	18+	230,664	126,316
Total	0-17	0	141,825
	18+	312,282	144,518
	Total	312,282	286,342

SDC 6. Number of patients per 1,000 per diagnosis based on physiotherapy claims of 2012 and of 2013. The prevalence of diagnosis 79 (7.47 and 7.31, respectively) is only partially visible.

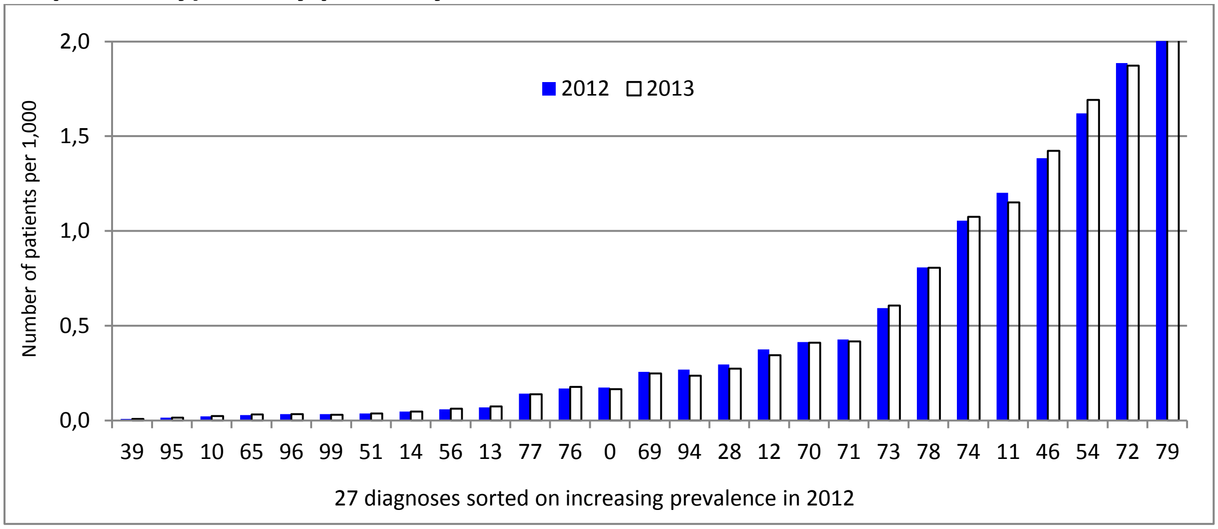

Supplement: Supplementary file 1 — Supplementary material 1 (PDF 714 kb) [file 10198_2017_874_MOESM1_ESM.pdf]
